# Minimally Invasive Mitral Valve Replacement in a Patient With a Giant Left Atrium

**DOI:** 10.7759/cureus.85423

**Published:** 2025-06-05

**Authors:** Laith Altawil, Mohammad Dayeh, Suhail Hammoudeh, Naser Jaber, Abdul-Hakim Dayeh

**Affiliations:** 1 Department of Cardiothoracic Surgery, Specialty Hospital, Amman, JOR; 2 Department of Cardiothoracic Surgery, Jordan University of Science and Technology, Irbid, JOR; 3 Department of Cardiology, Specialty Hospital, Amman, JOR; 4 Department of Anesthesia, Specialty Hospital, Amman, JOR

**Keywords:** giant left atrium, minimally invasive mitral valve surgery, mitral regurgitation (mr), mitral valve disease, mitral valve replacement (mvr)

## Abstract

A giant left atrium (GLA) is a challenging manifestation of advanced mitral valve disease, often necessitating mitral valve surgery via median sternotomy due to the anatomical distortion it causes. We present the case of a 50-year-old male patient with New York Heart Association (NYHA) class IV symptoms secondary to severe mitral regurgitation, found to have a left atrial diameter of 17.1 cm, to our knowledge, one of the largest ever reported. After declining sternotomy, the patient underwent successful minimally invasive mitral valve replacement. Despite significant anatomical challenges, the procedure was completed uneventfully through careful preoperative planning and specialized intraoperative techniques. While sternotomy remains standard in such complex presentations, advances in imaging, instrumentation, and surgical expertise are redefining procedural boundaries. This case demonstrates that minimally invasive mitral valve replacement can be safe, feasible, and effective even in patients with extreme atrial enlargement when performed by experienced teams.

## Introduction

A giant left atrium (GLA), typically defined as a diameter exceeding 80 mm, presents considerable challenges during cardiac surgery [1]. The estimated incidence of GLA is low, reported in less than 0.3% of patients undergoing mitral valve surgery [1]. It is frequently linked with chronic mitral valve disease and atrial fibrillation, often resulting in complications such as thromboembolism and hemodynamic compromise [1]. The marked atrial enlargement can distort anatomical landmarks, making surgical exposure and valve replacement technically demanding, particularly when attempting minimally invasive approaches. Consequently, median sternotomy remains the standard surgical route in such cases to allow adequate access [2].

## Case presentation

A 50-year-old male patient with longstanding mitral regurgitation presented with decompensated heart failure symptoms consistent with New York Heart Association (NYHA) class IV status, including progressive dyspnea, fatigue, and palpitations. He had no notable comorbidities or previous cardiac interventions. The patient had previously declined mitral valve replacement via sternotomy due to personal preference. There was no relevant family history and no psychosocial or genetic concerns affecting care.

On examination, the patient appeared dyspneic at rest and required assistance with ambulation. Cardiovascular assessment revealed a hyperdynamic apex beat and a loud holosystolic murmur best heard at the apex and radiating to the axilla. Pulmonary examination revealed bibasilar crackles. Signs of volume overload included bilateral peripheral edema and elevated jugular venous pressure.

**Figure 1:**
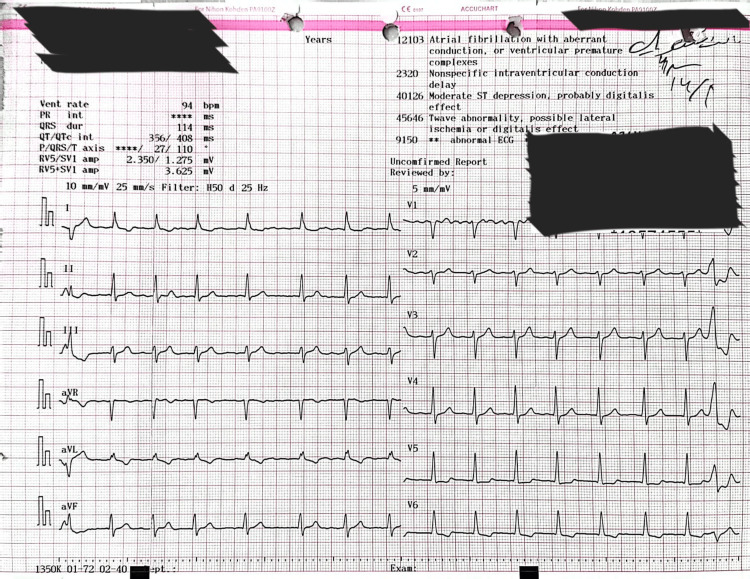
Electrocardiogram Preoperative 12-lead electrocardiogram showing atrial fibrillation with a controlled ventricular response, non-specific intraventricular conduction delay (QRS ~110 ms), and down-sloping ST depression in lateral leads suggestive of digoxin effect. These findings are consistent with chronic mitral regurgitation and a markedly enlarged left atrium.

**Figure 2:**
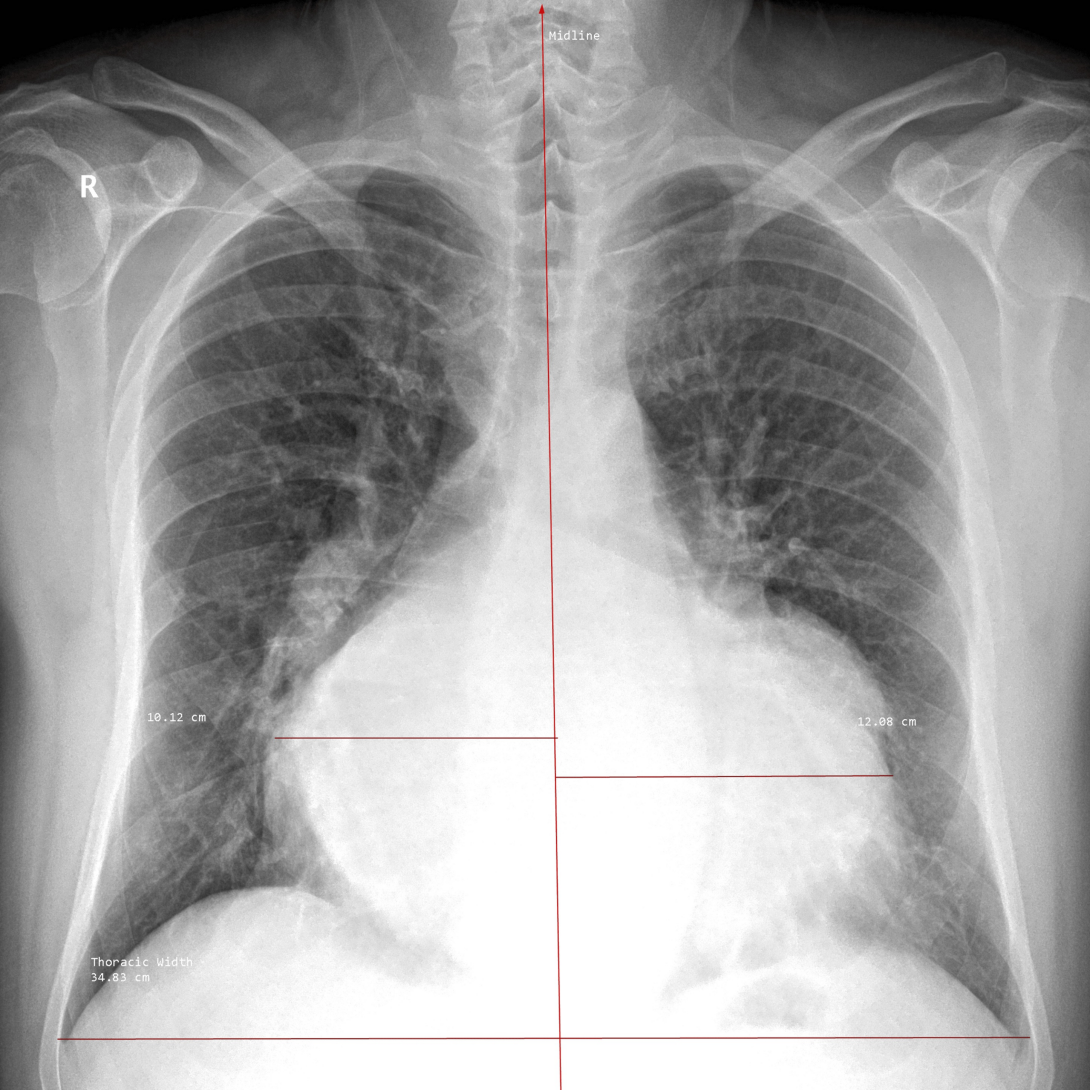
Chest radiograph Posteroanterior chest radiograph demonstrating marked cardiomegaly secondary to massive left atrial enlargement, with splaying of the carina and a cardiothoracic ratio (CTR) of 0.64.

**Figure 3:**
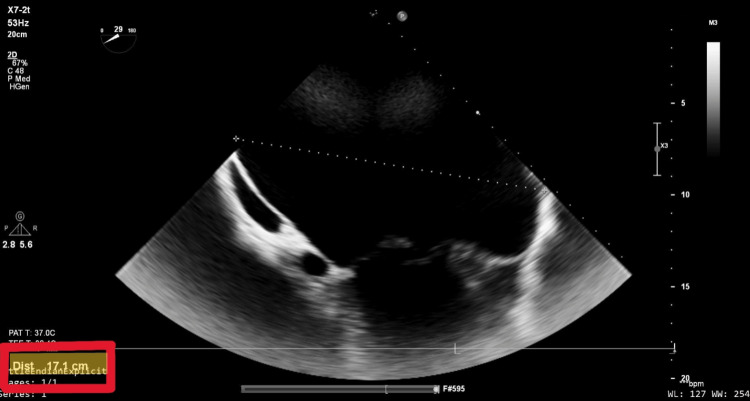
Transesophageal echocardiography Modified mid-esophageal view at 29°, selected to optimize visualization of the posterior left atrial wall. The anteroposterior diameter measures 17.1 cm, consistent with a giant left atrium. Adjacent cardiac structures are displaced, and typical chamber visualization is obscured due to the severe atrial enlargement.

Electrocardiography showed atrial fibrillation with a controlled ventricular response and nonspecific intraventricular conduction delay (Figure 1). Chest radiography revealed marked cardiomegaly, a cardiothoracic ratio of 0.64, and splaying of the carina, consistent with a severely enlarged left atrium (Figure 2). Transesophageal echocardiography (TEE) revealed a dilated left atrium measuring 17.1 cm in anteroposterior diameter, with preserved left ventricular systolic function. Severe mitral regurgitation was identified, with posterior leaflet restriction. Other cardiac chambers were partially obscured due to atrial displacement (Figure 3, Video 1). In addition, TEE confirmed the absence of thrombus in the left atrial cavity and appendage. Coronary angiography demonstrated normal coronary anatomy. Additional imaging was deferred due to the definitive echocardiographic findings and the urgency of the case.

**Video 1:** Preoperative transesophageal echocardiography Transesophageal echocardiography showing marked dilation of the left atrium and severe mitral regurgitation with restricted posterior leaflet motion and an anteriorly directed jet.

**Figure 4:**
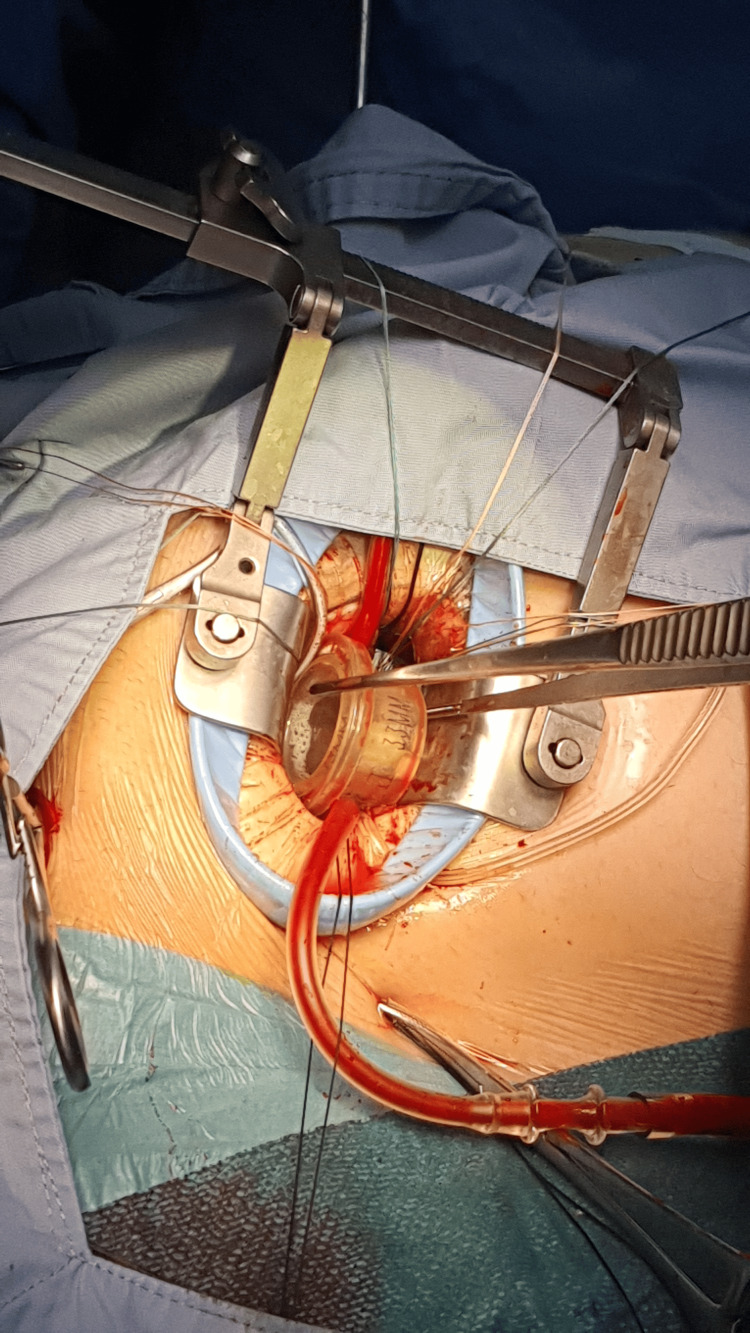
Intraoperative image of prosthetic valve implantation Intraoperative photograph demonstrating the implantation of a 33-mm Medtronic Hancock II bioprosthetic valve via a minimally invasive approach. A larger valve was selected to accommodate the markedly dilated mitral annulus.

The patient underwent minimally invasive mitral valve replacement through a 5-cm right anterolateral thoracotomy in the fourth intercostal space (Video 2). Femoral arterial and venous cannulation was used for cardiopulmonary bypass. The pericardium was opened anterior to the phrenic nerve, and stay sutures were placed to elevate the heart. The aorta was displaced anteriorly and leftward, but cross-clamping and cardioplegia delivery were completed without incident. Upon opening the left atrium, a markedly dilated right anterior pulmonary vein was noted, and the mitral valve was not immediately visible due to superior displacement. Valve exposure was achieved using interrupted pledgeted sutures to manipulate the annulus into view (Video 3). Intraoperative assessment revealed significant annular distortion, posterior displacement, and leaflet fibrosis, rendering the valve unsuitable for durable repair. Therefore, the decision was made to proceed with valve replacement rather than attempted repair. Subsequently, a partial resection of the A2 segment was performed, and a 33-mm Medtronic Hancock II bioprosthetic valve (Medtronic, Minneapolis, MN) was implanted successfully (Figure 4, Video 4). The left atrium was closed after thorough deairing, and the patient was weaned from bypass with moderate inotropic support. Intraoperative TEE confirmed excellent valve function with no paravalvular leak (Video 5).

**Video 2:** Minimally invasive incision This clip demonstrates the minimally invasive right anterolateral thoracotomy incision and includes live intraoperative footage, highlighting the markedly enlarged heart.

**Video 3:** Mitral valve visualization Clip demonstrating successful exposure of the mitral valve despite severe anatomical distortion caused by the markedly dilated left atrium.

**Video 4:** Bioprosthetic valve implantation Footage demonstrating the implantation of a bioprosthetic mitral valve via a minimally invasive approach, performed successfully despite significant anatomical distortion caused by the giant left atrium.

**Video 5:** Postoperative transesophageal echocardiography Post-implantation transesophageal echocardiography demonstrating successful deairing and excellent prosthetic mitral valve function, with no evidence of paravalvular leak or structural abnormalities.

A formal atrial reduction was deferred because the patient’s atrium, although massive, was hemodynamically silent, and the minimally invasive approach precluded safe plication.

The postoperative course was uneventful. The patient was discharged in stable condition on postoperative day four. At follow-up, he reported significant improvement in dyspnea and functional capacity. Transthoracic echocardiography confirmed good prosthetic valve function with no complications. The patient remained well without readmission, arrhythmias, thromboembolism, or wound-related issues.

## Discussion

Surgical management of GLA remains a formidable challenge, particularly in patients with chronic mitral regurgitation and atrial fibrillation, though current guidelines from the American Heart Association (AHA)/American College of Cardiology (ACC) and the European Society of Cardiology (ESC)/European Association for Cardio-Thoracic Surgery (EACTS) emphasize individualized decision-making, incorporating anatomical complexity, disease severity, institutional expertise, and patient preference [3,4]. Despite this flexibility and to the best of our knowledge, minimally invasive mitral valve surgery (MIMVS) for patients with a GLA has rarely been reported, highlighting the limited literature available on this specific surgical approach in such anatomically challenging cases and affirming that conventional median sternotomy remains the favored approach in most cases involving significant left atrial enlargement due to the technical demands of exposure and repair, which explains the paucity of utilizing the minimally invasive technique in the surgical management of GLA in literature. 

Several studies have demonstrated that MIMVS is associated with shorter hospital stays, reduced perioperative bleeding, and lower rates of wound infection compared to conventional sternotomy [5, 6]. In light of these advantages, literature reports have highlighted the growing feasibility of MIMVS, even in anatomically complex cases such as left atrial enlargement. This expanding applicability is supported by advancements in transesophageal echocardiography, meticulous preoperative planning, the adoption of high-definition endoscopic instrumentation, and the increasing experience of surgical teams, all of which have significantly enhanced intraoperative visualization, procedural precision, and overall outcomes [7, 8]. Notably, Babilek et al. described a successful case of MIMVS with concurrent atrial plication in a patient with a left atrial diameter of 9.1 cm, demonstrating significant postoperative size reduction and clinical improvement [9].

Our case further contributes to this evolving paradigm by demonstrating the successful performance of minimally invasive mitral valve replacement in a patient with a left atrial anteroposterior diameter of 17.1 cm, among the largest reported in the literature. The patient had previously declined sternotomy, and this preference guided the choice of MIMVS. Intraoperatively, the team encountered substantial anatomical distortion: the mitral valve was obscured due to superior displacement, the right anterior pulmonary vein was dilated to 4 cm, and the aorta was anteriorly displaced, complicating standard exposure and cannulation techniques. Nevertheless, careful surgical planning, strategic use of pledgeted sutures to manipulate the valve annulus into view, and precise bioprosthetic valve implantation enabled a successful outcome without complication.

This reinforces that, with careful planning and expert teams, MIMVS can be safe and reproducible, even in cases traditionally managed via sternotomy. However, it also illustrates a practical limitation: such technically demanding procedures may not be easily replicable across all institutions and require significant surgical expertise, institutional support, and dedicated instrumentation.

As surgical capabilities continue to evolve, it is increasingly important to reconsider traditional limitations to minimally invasive strategies, ensuring that patients are offered the most appropriate and individualized approach based on current best practice.

## Conclusions

This case challenges conventional surgical paradigms by demonstrating the feasibility of minimally invasive mitral valve replacement in a patient with one of the largest reported GLAs. It highlights that minimally invasive mitral valve replacement can be safely and effectively performed in anatomically complex scenarios such as GLA when guided by careful planning, surgical expertise, and institutional experience. Furthermore, this case supports the expanding role of minimally invasive approaches in complex cardiac surgeries and encourages their consideration even in situations traditionally managed by median sternotomy.
